# Data fusion for automated non-destructive inspection

**DOI:** 10.1098/rspa.2014.0167

**Published:** 2014-07-08

**Authors:** N. Brierley, T. Tippetts, P. Cawley

**Affiliations:** Department of Mechanical Engineering, Imperial College London, London SW7 2AZ, UK

**Keywords:** data fusion, defect detection, automation, receiver operating characteristic

## Abstract

In industrial non-destructive evaluation (NDE), it is increasingly common for data acquisition to be automated, driving a recent substantial increase in the availability of data. The collected data need to be analysed, typically necessitating the painstaking manual labour of a skilled operator. Moreover, in automated NDE a region of an inspected component is typically interrogated several times, be it within a single data channel due to multiple probe passes, across several channels acquired simultaneously or over the course of repeated inspections. The systematic combination of these diverse readings is recognized to offer an opportunity to improve the reliability of the inspection, but is not achievable in a manual analysis. This paper describes a data-fusion-based software framework providing a partial automation capability, allowing component regions to be declared defect-free to a very high probability while readily identifying defect indications, thereby optimizing the use of the operator's time. The system is designed to applicable to a wide range of automated NDE scenarios, but the processing is exemplified using the industrial ultrasonic immersion inspection of aerospace turbine discs. Results obtained for industrial datasets demonstrate an orders-of-magnitude reduction in false-call rates, for a given probability of detection, achievable using the developed software system.

## 1. Introduction

Non-destructive evaluation (NDE), also termed non-destructive testing, is a field of applied physics and engineering of significant industrial importance, both for quality assurance in high-value manufacturing, for example in the aerospace industry, and maintenance of plant, for instance, in power generation. NDE encompasses a wide range of sensing techniques, from thermography to radar and ultrasonics to radiography [1], and there is some overlap with the fields of both medical imaging [2] and remote sensing [3].

The particular choice of technique is very application specific, dependent not only on the material of the component to be examined, but also on the nature of the defects one wishes to guard against. It can in fact be very advantageous to use more than one sensor system, with the potential to improve coverage, inspection speed and/or sensitivity.

There are significant costs associated with conducting industrial NDE. Staff time, inspection equipment and consumables, and outage or production time, for mid-manufacture and in-service inspections, respectively, all contribute. Given these costs of inspection, NDE is only applied where the costs of failure and component replacement are very high, but the probability of a defect necessitating the replacement of a part is low. For inspection prior to operation, during manufacture, NDE can be used to place an upper bound on imperfections present, enabling longer service lives to be guaranteed, with important implications for operating costs. Because NDE is important for managing the risk to life, limb and property associated with highly load-bearing components, in many industries the use of NDE is mandated by regulation, often in response to a catastrophic failure [4].

One general inspection approach that industries have been increasingly adopting is the automation of the data collection in an inspection. Automated inspection involves a mechanical scanning system moving one or more sensors across the component systematically while data are collected. Such automation is attractive to industry as the variability in the data collection is significantly reduced compared with manual approaches. The systematic nature of the scan ensures coverage and consistency across different parts, and reduces the scope for human error to compromise the inspection. The higher scanning speeds and positional accuracy achievable by mechanical means also allow more data to be collected from a part in less time than in traditional approaches, especially if multiple sensors are deployed simultaneously. In principle, more data bring the promise of greater defect sensitivity. Automated inspection also relieves staff of the most tedious work, often in cramped or otherwise uncomfortable and potentially dangerous environments. Moreover, compared with manual ultrasonic testing (UT), for example, a complete and permanent record of the inspection can be obtained, suitable for off-line analysis, easy reporting and insurance purposes.

**Figure 1.**
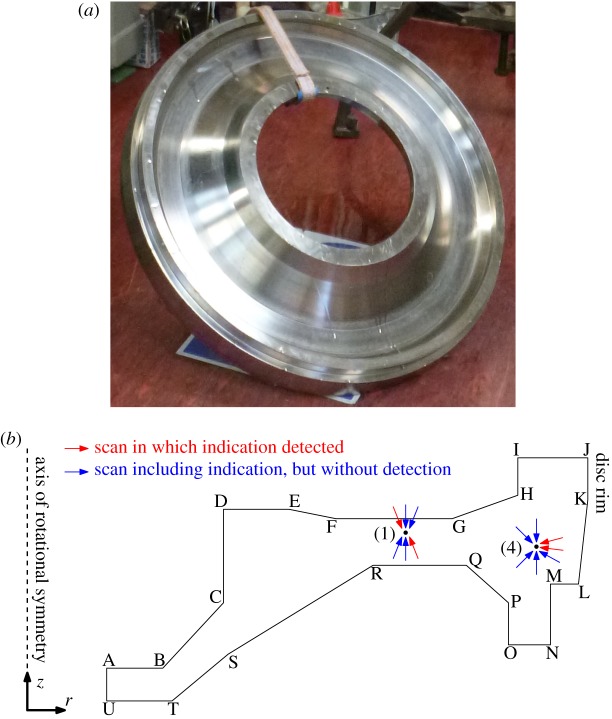
A titanium aerospace jet engine disc to be inspected in its mid-manufacture stage. (*a*) A photo is presented, showing the underside of the disc, clearly revealing the different surfaces of the rectilinear cross section that are to be scanned. The disc is approximately 90 cm in diameter. *Photo taken at Rolls-Royce site.* (*b*) The corresponding schematic cross section is shown. Each individual surface is identified from the bounding two letters, and each surface is scanned at at least one angle. Each scan then is identified by the surface insonified and the probe angle to the surface normal. The points marked 1 and 4 correspond to the known indications of seeded defect disc 5, a Rolls-Royce test piece used in the later results. For these two points, the diagram indicates from which directions the indications might be seen, and in which scans these indications are detected by the current data analysis procedure. *Diagram derived from one provided by Rolls-Royce.*

The example inspection that serves as the source of data in this paper concerns the inspection of titanium aerospace jet engine disc forgings, as used at Rolls-Royce. The integrity of discs is essential to the safety of jet engines, because the kinetic energy of a disc when the engine is under load is so high that failure containment by the engine housing is impossible. Given access constraints in an assembled engine, discs are only inspected during manufacture and then assigned a service life, after which the part is replaced. The inspection therefore seeks to identify any inclusions or similar imperfections in the disc larger than the tiny defect population size assumed in lifing; such larger defects could serve as crack initiation sites and then lead to failure before the end of the calculated service life. Because the final shape of these discs is extremely complicated, including for instance slots to hold blades along the outer circumference, discs are inspected in a mid-manufacture stage when the disc has a rectilinear cross section. A photograph of a typical disc is shown in figure 1*a*, next to the disc cross section in figure 1*b*. The relatively simple shape, at least compared with the final product, greatly simplifies the inspection as there are fewer interfering reflection and diffraction effects.

The discs are inspected in a water-filled immersion tank system (D. Wright 2010, private communication). A disc is lifted onto a turntable that then spins while a manipulator arm above moves a single pulse-echo ultrasonic probe in a radial plane. The probe is moved to scan each surface of the disc rectilinear cross section (figure 1*b*) in turn, with the probe taking an axial and/or radial step after every turntable revolution. In fact, each surface is scanned three times; normal to surface, and at +5^°^ and −5^°^ to the surface normal, in a radial plane. Half-way through the inspection, the disc is manually flipped over to allow access to the other side of the disc. Before the start of the disc scan, the probe is calibrated using a set of flat-bottom hole test pieces down the side of the tank. A distance amplitude correction (DAC) is computed and applied so that all recorded data already include a compensation for beam spreading and attenuation [5].

Current data analysis and defect detection relies on a global, fixed amplitude threshold set at −18 dB down from the flat-bottom hole calibration. Any signal that reaches or exceeds this threshold is investigated manually (D. Wright 2010, private communication). Unfortunately, there are many false calls as the scan is complicated by spurious high amplitude signals. Such signals can be the result of microstructural noise, given that these titanium parts tend to contain neighbourhoods of large, closely aligned grains [6,7], and surface machining imperfections, for example. Real defects are extremely rare, but a significant number of discs do not pass the inspection and at least need some further work prior to further manufacturing stages.

The Rolls-Royce inspection system does have the benefit of several seeded defect discs, containing realistic defects after having been forged from contaminated billet. These may be used to test detection performance: data from such a disc are used as the basis of the results section, considering the two known indications indicated in figure 1*b*. This figure also reveals how the described amplitude threshold detection system only identifies the known indications in a small fraction of the available scans that had the opportunity to detect at those locations.

The saved data from a single disc are multiple gigabytes in size, containing millions of A-scans, so that the data volumes collected are overwhelming to a human operator and place significant demands on computer hardware. The set amplitude threshold used for detection is arbitrary in the sense that it is based on idealized target reflectors, and it is unlikely that any real defect would resemble one of these. Moreover, such a threshold is unable to take into account variations in the microstructural noise across a component, strong return signals from the vicinity of geometric component features, or the amplification of electronic noise due to higher effective gain at greater A-scan depths. Related to this, the high number of false calls experienced due to signals exceeding the set amplitude threshold slows down the inspection considerably and is draining for a human operator to deal with. Lowering the amplitude threshold used for detection to enable the detection of smaller indications, and hence for instance longer service life ratings or maintenance intervals, would make the number of false calls too high to manage. No meaningful, systematic attempt to consider signals from different scans or channels simultaneously is made, even when these relate to the same sample volume. Furthermore, humans are fundamentally poor at assessing random behaviour and correlations [8], compromising both the assessment of signals in high noise environments and across channels, if this were attempted. Overall, these shortcomings mean that the inspection reliability is at the very least not as good as it could be given the available data. The software system described here seeks to address all the identified problems to allow automated inspection to reach its full potential. (Throughout this paper, we use ‘software system’ to mean the whole program that takes as-recorded inspection data as its input and ultimately returns detection results.)

This paper proceeds by providing an overview of the context of the data fusion approach adopted. Then the novel probabilistic data fusion system, featuring a semi-parametric local data model and a discretization-compensated consensus test, is explained in detail. The results section then demonstrates the performance of the system on a real, industrial dataset input. Finally, we offer some concluding remarks.

## 2. Data fusion context

This section provides an introduction to data fusion before we develop a general approach for the probabilistic combination of signals in an identified region of the component, containing spatially coincident sections of arbitrary amplitude fields, from different data channels whose domains intersect that region. The intention is for such a probabilistic evaluation system to be used as a data fusion detector (DFD). This is not designed to be fully automatic or offer any kind of defect classification capability, in contrast to several detection approaches reviewed below. Rather the DFD is expected to provide a skilled human operator with a sequential analysis interface: the software will suggest component regions for further manual review ranked by a displayed indication severity metric computed based on the data fusion. In principle, the software will continue returning component regions until the set of returned regions makes up the entire component. However, in practice the human operator will review the first few, and having satisfied him-/herself using current indication assessment procedures that the most serious indications are benign, will terminate the analysis. Therefore, this interface leaves all sentencing decisions to the operator but provides an advanced analysis aid to focus the operator's attentions on the component regions where his or her skills are best applied. In addition to significant time (and hence cost) savings, such a system offers improved inspection reliability, both because the data fusion is more rigorous and comprehensive than any simplistic analysis scheme and because there is less scope for fatigue and human error.

### (a) Signal mixture model

**Figure 2.**
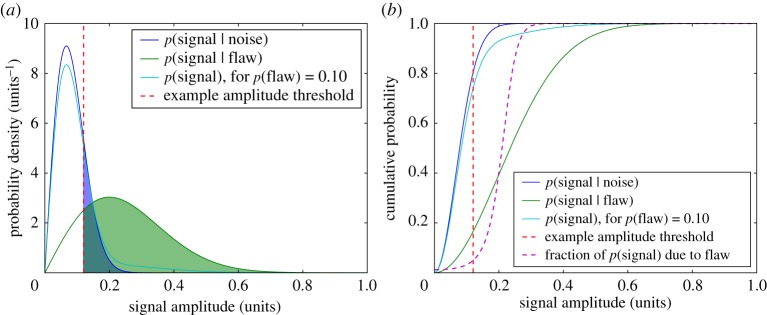
A signal mixture model. In (*a*), probability density functions (p.d.f.s) are shown for the noise distribution, the flaw distribution and the overall signal distribution, formed as a weighted sum from the two other distributions given the flaw prior. Plot (*b*) shows the corresponding cumulative distribution functions (c.d.f.s), and the fraction of the signal explained by the flaw distribution. Both plots also feature an example of an amplitude threshold, by which signals are traditionally taken to originate from a flaw if they fall above the threshold amplitude. For a given amplitude threshold, the probability of detection (POD) and probability of false alarm (PFA) may be computed by integrating the p.d.f. *p*(signal|flaw) and *p*(signal|noise), respectively, from the threshold up as shaded in (*a*)—and so these metrics may be read off (*b*).

The widely used signal mixture model offers a simple (single-channel) representation of the problem that we seek to address and is illustrated in figure 2. The basic premise is that the signal recorded is described by a distribution that is the weighted combination of distributions for the noise and for a flaw of a specified type. These component distributions, expressed in terms of conditional probabilities (e.g. *p*(signal|flaw) is the probability distribution of signal given that it is caused by a flaw), are not available in practical data analysis. The objective of the analysis is to determine whether a particular recorded signal is the result of noise, or more importantly, a flaw. The traditional approach is to threshold the signal amplitude, declaring signals that fall above to be due to a flaw and below due to noise. As shown in the figure, POD and PFA may be computed for a given threshold, and sweeping this threshold also allows the receiver operating characteristic (ROC) to be built up [9–11].

The approach described for identifying flaw signals is in many ways unsatisfactory. Firstly, even though there are algorithms for the choice of threshold value [12], this choice is essentially arbitrary, and in general the selection is not probabilistic, with little regard for the consequences in terms of POD and PFA. Secondly, there is the fact that this a form of binary classification, when a fuzzy class membership [13,14] would allow the uncertainty to be taken into account and be more helpful for ranking signals—consider, for example, replacing the step-function-like threshold with a smoothly varying curve such as the fraction of the signal due to the flaw distribution shown in the cumulative distribution mixture model plot figure 2*b*. Additionally, focusing on raw amplitudes neglects a lot of information about the data acquisition that has the potential to improve the chances of a correct classification. We therefore desire a classifier system that effectively expresses signal membership of the noise class as a probability, in the [0,1] interval. In view of the desired output from the detector, and the wish to minimize the number of assumptions required, the classifier to be designed is of the *one-versus-rest* form, where no attempt is made to distinguish between the signals from different types of flaw [15].

### (b) Multi-channel data

In addition to the single-sample evaluation considerations described so far, we wish to exploit the fact that in automated inspection each part of the component is typically interrogated several times. The benefits of data fusion are both intuitively obvious and well-documented [16]. Therefore, the spatially coincident sections of diverse amplitude fields, from different data channels whose domains intersect a region of interest, should be combined in a logical and general way to aid the region's classification. Different types of combination, depending on field modality, need to be considered. Firstly, there is the combination of field sample values within a single channel, especially relevant as any real defect is expected to be visible in more than one probe position even within a single channel, given the dimensions of any physical defect and the probe's beam spread, for example. Then there is the combination of channels of differing modality (e.g. using different types of ultrasonic probe, or even using a completely different technology, such as eddy-current), acquired during one and the same inspection. Finally, there is the incorporation of field samples from a procedurally identical, but previous, inspection, which then moves the processing into the realm of baseline subtraction [17,18] and change detection [19].

Data fusion literature defines different levels at which fusion may take place: broadly, data-level, feature-level and decision-level [20,21]. The first refers to combining raw amplitudes, the second to extracting compatible features and combining these, and the last, the highest level, relates to combining the decisions reached by analysing different data channels independently. While all three levels have applications, in general, higher levels result in coarser results than lower level processing, but permit savings in data handling volumes. There is also one critical restriction to bear in mind in deciding at which level to undertake fusion: data-level processing requires amplitude values to be compatible—for example, subtracting amplitudes generated by sensor systems based on different technologies is largely meaningless. Mono-modal datasets, that is to say of identical type (for our purposes, identical ultrasonic probe specification and angle—not all ultrasonic channels are considered to be one modality), are compatible after registration and may therefore be processed at the data-level, in raw amplitude form. Conversely, multi-modal datasets, for example containing data from shear and near focus compression ultrasonic probes, are not compatible and must be fused at a higher level, preferably the feature-level. We apply a hybrid scheme, using data-level processing where possible and moving to the feature-level to overcome differences in channel modality.

### (c) Possible approaches

There is a vast array of data fusion algorithms, especially in the context of military applications, such as the identification of ground units from airborne infrared and radar sensor systems [22]. However, a more limited range has so far been trialled on NDE data, in part because some of the most complex considerations in military uses are not relevant—surveys are provided by Zheng *et al*. [21] and Gros [23]. Prominent possibilities include use of fundamental arithmetic operators such as addition, operating on raw amplitudes or extracted features [24–26], *Bayesian* inference [25,26], *Dempster–Shafer* fusion [16,25–27] or artificial intelligence (AI) [28,29]. However, the approach taken here is based on classical inference [22,23,30], largely as this makes fewer assumptions and has greater intuitive appeal than alternatives, and should prove adequate for our *one-versus-rest* classification scenario. The simplicity of classical inference is especially appealing as [16] suggests that the simple fusion methods already offer good performance, and that while complex techniques may give further gains, these have to be weighed up against increased computational effort and the need to make further assumptions.

Classical inference is based on hypothesis testing: a null hypothesis *H*_0_ is defined alongside the antithetical alternative hypothesis *H*_1_. A probability is assigned to a random variable according to how likely the value observed, *x*_*a*_, or one more extreme (i.e. higher), is to occur under the null hypothesis *H*_0_ data model for the random variable. This is the *p*-value of the random variable under the distribution, equal to the integral of the p.d.f. over the interval $\left(x_{a},\infty \right)$, i.e. *P*(*x*_*a*_|*H*_0_)=1−*P*_c.d.f._(*x*_*a*_). The *p*-value may also be expressed in terms of the survival function, *P*_s.f._(*x*_*a*_), where *P*_s.f._(*x*)=1−*P*_c.d.f._(*x*), which then gives the *p*-value directly as a function of the random variable. The definition of a *p*-value means that this statistic can serve as a measure of the extent to which the observed value can be explained by the data model, which for our purposes will essentially be a noise model. Usually, a significance level, typically *α*=5%, is used to threshold the *p*-value: if the computed value lies above the threshold, this is deemed not to refute the null hypothesis, *H*_0_, whereas a value below is interpreted as a rejection of *H*_0_ (e.g. ‘noise’) and the alternative hypothesis *H*_1_ (e.g. ‘non-noise’, i.e. ‘flaw’) is deemed to apply. Note that the significance level in effect specifies the expected false-call rate [22,23,30].

### (d) Registration

The alignment of available data channels to a common coordinate system, known as registration, is a critical pre-processing stage for any subsequent comparison or combination of data from different channels in a joint analysis. Registration is important in many fields, but most prior work concerns (two-dimensional) image registration [31]. A substantial overview of the subject is provided by Zitova & Flusser [32], in which the four major steps in most registration procedures are identified: feature detection, feature matching, transform model estimation and image transformation. Another survey of techniques, but drawn from medicine, is provided by Maintz & Viergever [33]. While there are parallels to medical imaging in this work, especially in terms of the dimensionality and potential multi-modality of the data [34], NDE test subject variability is low and the types of possible distortions limited compared with the human body [35,36]. The system developed and adopted by the authors is described in detail in [37–39]. Key features of this registration framework include a physical model of the data acquisition that attempts to describe all conceivable benign distortions of the data and the use of a multi-objective optimization [40], allowing alignment and hence positional uncertainty to be assessed, with consequences for the later data fusion. In the case of the disc inspection application, registration accuracies of 2–3 mm have been consistently achieved, over a range of test displacement magnitudes [38]. All sections that follow assume that registration of the different data channels has already been completed.

## 3. Data fusion method

As data-level processing is possible for data from a given channel modality, within each individual channel useful processing steps specific to each modality, for instance, taking into account different probe effects, may be applied prior to cross-channel fusion. The processing may take the form of image processing algorithms, such as noise filtering or the Synthetic Aperture Focusing Technique (SAFT) [41]. An implementation of the SAFT algorithm for the sort of single probe data considered here was developed, providing one possible way of incorporating a beam model in each channel. If there are data from an equivalent channel in a previous acquisition available, accurately registered to the data in the current channel, then this within-modality processincg allows the historic data to be incorporated through baseline subtraction. However, in practice for the initial application these refinements were not employed and the individual channels only underwent basic filtering operations. Having completed all channel-specific processing on the data channels independently, feature-level fusion becomes necessary to combine fields from different probe types. Given that the sought output of the data fusion processing is a probability, the feature chosen for this is a set of probabilities.

### (a) De-correlation

The choice of how to process amplitude fields in terms of probabilities was inspired by functional magnetic resonance brain imaging (fMRI) [30]. In this area of medical research, multi-dimensional fields of MRI data are analysed to identify ‘activated’ regions of the brain under various experimental conditions, to make deductions about brain function. The statistics developed and applied in this field also permit for example complex models to be hypothesis tested in the presence of confounding effects. One of the problems the fMRI literature describes in attempting to apply statistics to amplitude fields is that statistical methods generally require independent, identically distributed (i.i.d.) data samples [42]. The various amplitude field samples available will not be of this sort, as within each channel there will be significant correlations in samples at proximate locations, which mean the samples cannot be considered independent. It should be stated, however, that samples drawn from different data channels are considered statistically independent for the purposes of this analysis, so what follows in this section refers to samples from a single channel.

The described correlations between proximate locations vary in magnitude and causes with direction. Thompson *et al.* [43] showed that in the probe displacement direction(s), the extent of overlap of the insonified volume for adjacent probe locations and the sample microstructure are the main parameters controlling spatial correlations. The former varies with depth, controlled by the beam spread of the probe and the probe step size between A-scan acquisitions, while the scale of the latter compared with the inspection wavelength determines the nature of the interaction of the sound with the medium and specifies how much averaging of grain contributions occurs over an insonified region. On the other hand, in the time-axis or depth direction the pulse length (or equivalently, bandwidth) together with the A-scan sampling frequency and material microstructure govern the extent of sample correlation. Additionally, subsequent signal processing can introduce further sample dependencies. For example, envelope detection will necessarily add considerable correlations to samples along the time-axis. Such correlation considerations also have direct applications, in the specification of optimal inspection sampling procedures, to ensure coverage is assured and independent data maximized but redundant data minimized [44]. In practice, for confidence in the detection of small reflectors that fall exactly between two sample locations and so as to preserve the capability of averaging out random noise without the loss of independent information, slight oversampling is desirable.

To obtain approximately i.i.d. samples, an empirical approach is taken that avoids the need to untangle the various correlation contributions and compensate for them individually. First, the extent of sample dependence is estimated by computing correlation lengths of the samples in different directions, taking care to exclude high amplitudes from prominent reflectors, as one of the defining features of signals from reflectors of interest is that they have a correlation differing from the background. In this work, the correlation length is defined as the full-width at half-maximum of the average autocorrelation function of the data in each index-space dimension [30]. Then, borrowing from fMRI data analysis, the sampled field of voxels (individual data values, each assigned to a small region of three-dimensional space), which is itself a discrete representation of a continuous physical field, is further discretized. The scale of discretization in each dimension is given by the maximum of the correlation length in that dimension and the estimated registration error, such that a poor registration accuracy will compromise the resolution of the subsequent fusion processing. The formed multi-voxel chunks of space are referred to as resolution elements, or *resels*, each representing a single, approximately independent sample [30,45]. Especially given for instance the need to align *resel* boundaries with index-space steps, it is accepted that this will give imperfect de-correlation, but the current implementation appears sufficient for present purposes. The value assigned to each *resel* is computed from the contained voxel values, reducing these to a single representative value—here the maximum is used. This quantity foregoes the reduction in random noise that might be possible by averaging, but it has the advantage of being conservative and compatible with traditional perspectives. At first, the reduction of multiple voxel values within a *resel* to a single number may seem like gratuitous discarding of data, but in theory this procedure will merely discard sample values that provide only duplicate information.

**Figure 3.**
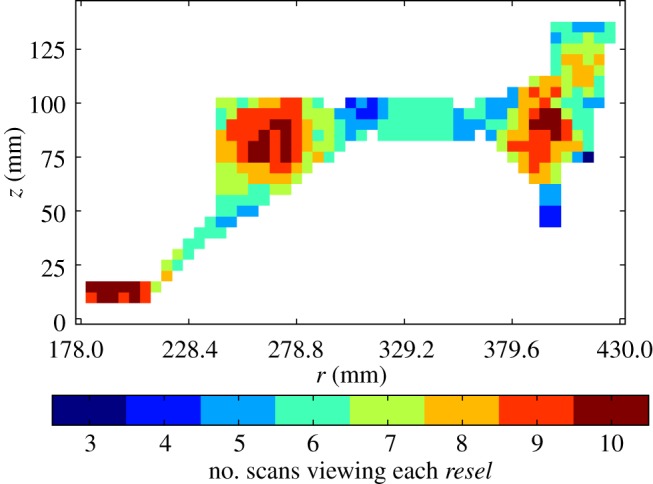
The coverage map for the disc inspection, counting the number of times each *resel* in a radial cross section of the component is insonified across all scans collected. The disc axis of rotational symmetry is the *z*-axis, and the radius *r* is measured from this. Note that every *resel* in the cross section is scanned at least three times, and some are viewed up to 10 times. Thus, there are significant opportunities for data fusion.

For the disc inspection, the *resels* used are approximate cubes with 5 mm edges. A coverage map for the inspection, illustrating these *resels* and counting the number of times each *resel* in a radial cross section of the component is insonified across all scans collected, is presented in figure 3. The plot shows that every *resel* in the cross section is scanned at least three times, and some are viewed up to 10 times, implying significant opportunities for data fusion. Having reduced the amplitude field of each channel to a large number of approximately independent amplitude samples, the next step is to combine those *resels* within- and cross-channel falling inside a defined spatial region of interest to ultimately yield a single, useful probability for that region.

### (b) Combination considerations

For (feature-level) fusion of *resel* values identified as falling within a chosen spatial region of interest across all data channels, the *resels* must be expressed in terms of a compatible feature. The sought output of the data fusion processing is a probability, so probabilities are the feature of choice, such that the feature-level fusion may be performed using relatively conventional statistics, operating on nominally i.i.d. inputs. The probabilities used must represent the significance of each *resel* within its channel, so that the feature is invariant to differences in amplitude scale between channels, for example.

Basic classical inference introduced previously may be extended to multivariate data. In the presence of *k*-independent tests, all the random variables recorded may be converted to *p*-values, *q*_1_,…,*q*_*k*_ on the basis of null hypotheses *H*_01_,…,*H*_0*k*_, and examined from the perspective of a universal, or family-wide, null hypothesis *H*_0_, which states that all of *H*_01_,…,*H*_0*k*_ are true. The corresponding universal alternative hypothesis *H*_1_ is that at least one of the component alternative hypotheses is true [46]. Defining a universal null hypothesis is common practice in biological meta-studies [47] and fMRI [30]. For the purpose of the data analysis here, this procedure provides the justification for converting the *resel* amplitude values to *p*-values and then evaluating these together. The data model to apply in the conversion to *p*-values is developed in the next section. Having incorporated channel-specific effects into the calculations of these probabilities, the *p*-values, regardless of channel of origin, are now compatible features, and may be combined together. This collection of *p*-values is then finally reduced to a single output probability, as discussed in §3*d*.

### (c) Data model

#### (i) Null hypothesis

The objective of the data model is to facilitate the conversion of *resel* amplitudes to *p*-values. The critical characteristic of *p*-values is that under the null hypothesis, these values are uniformly distributed over the interval [0,1]. This property follows from the definition of the c.d.f. (and hence survival function, s.f.): samples drawn from an arbitrary p.d.f. will be mapped to U(0,1) by the c.d.f. (or s.f.) of the distribution. An alternative explanation is that to generate random variable samples from an arbitrary p.d.f., samples from U(0,1) may be passed through the distribution's inverse c.d.f., mapping the U(0,1) samples to the arbitrary distribution's random variable space—so the reverse transformation is provided by the c.d.f. The null hypothesis assumed for each *resel* is that it is a random sample from the data model distribution, which must be defined.

#### (ii) Local data modelling

**Figure 4.**
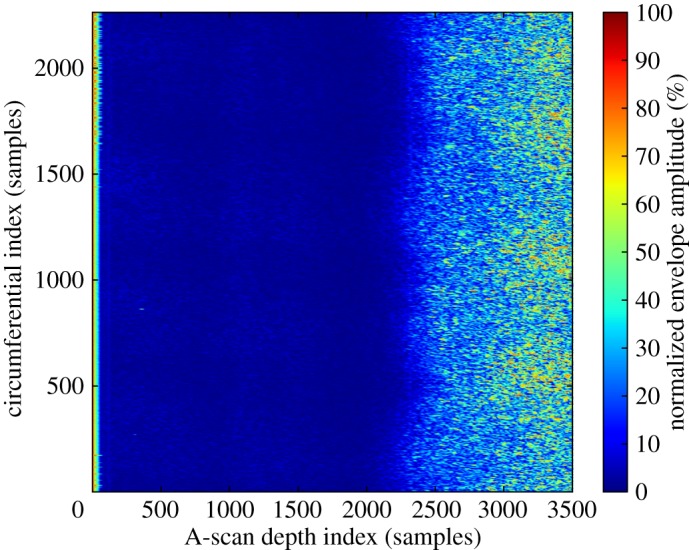
DAC B-scan image corresponding to data recorded during one rotation of a turbine disc, with the probe scanning radially inwards. On the left-hand side, the front wall echo is visible—the water path is not shown. On the right, at higher A-scan sample indices, corresponding to longer flight times and greater depths, there is a marked increase in coherent noise. See electronic supplementary material for data.

Attempts to define a global model for the data of each channel were found to be unsuitable in practice as for many data channels there are systematic variations in noise behaviour across a scan. The problem is illustrated in figure 4, where over the length of every A-scan there is a transition from a low noise region to a very noisy region, so that the complexity of these data cannot meaningfully be represented by a single distribution that implicitly assumes some degree of uniformity.

Therefore, the decision was made to use local data modelling and exploit symmetries of the scan to define an environment of similar *resels* for each *resel* location. In the disc inspection, rotational symmetry features prominently and it is reasonable to assume that *resels* in the same ring about the axis are in the same environment. Importantly, large circumferential (spanning a substantial fraction of the circumference) defects are unheard of so the reduced sensitivity to signals of such defects is not a problem. Assessing each *resel* in terms of its local environment in this way can be thought of as normalizing these amplitude samples circumferentially, with the chosen distribution within each hoop mapping the amplitudes to the interval [0,1]. Note that this scheme is extremely general, and can be adapted to suit a range of component geometries and inspection types. Even in the case of highly complex geometries lacking obvious symmetries, the same principles can be applied by considering each *resel* in terms of the set of *resels* at equivalent positions across a population of practically identical parts.

#### (iii) Discrete data

In considering how to construct a data model distribution from a collection of local *resel* amplitudes, it is necessary to bear in mind that this collection of amplitude samples is discrete, both by having been quantized during the digital recording process by an analogue-to-digital converter, and by virtue of the fact that only a finite, integer number of samples is available. Both these contributions are almost universally applicable to the processing of digital data, though in some cases the level quantization may be lost by subsequent data processing, for example, performing envelope detection on digitally recorded full-waveform A-scans. These forms of discretization can invalidate the application of statistics that at least implicitly assume continuous inputs.

**Figure 5.**
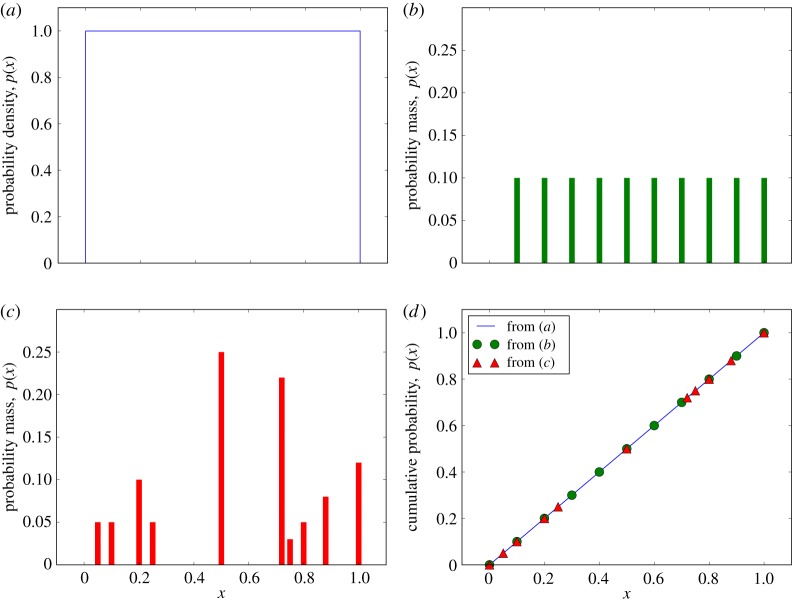
Uniform distributions over [0,1] interval, U(0,1), as a function of a random variable *x*. In (*a*), the continuous p.d.f. is shown, (*b*) shows the probability mass function (p.m.f.) of a possible discrete version of the distribution with 10 quantization levels, and (*c*) presents an alternative p.m.f. for a different set of 10 discretization levels. Plot (*d*) provides the c.d.f. for the previous three distributions: this straight-line c.d.f., between the origin and (1,1) is common to all versions of the uniform distribution over the interval considered, as indicated by the way the points derived from (*b*) and (*c*) map onto the line derived from (*a*).

As has been established, the uniform distribution (of *p*-values) over the interval [0,1] is central to hypothesis testing. The continuous version of this distribution is shown in figure 5*a*, and this is the only possible p.d.f. However, considering discrete versions of U(0,1), it soon becomes apparent that there exists an infinite number of distinct possibilities, defined by the discretization levels available. For instance, [0,1] may be split up into any integer number of levels of regular spacing, as exemplified in figure 5*b*, where there is still a strong similarity to the continuous version. However, the interval may alternatively be split into an irregularly spaced set of levels, demonstrated in figure 5*c*, where the visual similarity to the continuous distribution is practically lost. However, the form of the c.d.f. shown in figure 5*d*, with points lying on a straight line between the origin and (1,1), is common to all possible versions of U(0,1), continuous or discrete, and so this will be used as the defining property of any *p*-value distribution considered.

#### (iv) Semi-parametric model

**Figure 6.**
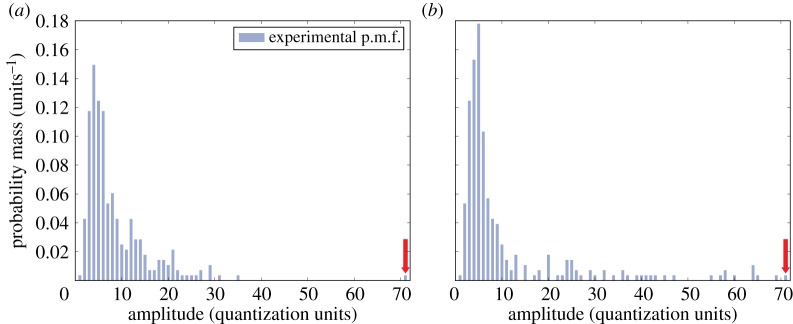
The amplitude p.m.f.s found in two rings of *resel* data. Note that the amplitude values are quantized due to the digital signal recording used and the probability mass values are quantized by virtue of the fact that these are experimental distributions, built up from the limited number of samples available in a ring of *resels*. While the sample at the highest amplitude (indicated by arrow) is both identical in amplitude and of the same frequency across the two plots, differences between the remaining distributions should be reflected by the data model's assessment of those samples. Intuitively, the likelihood that the highest amplitude sample can be explained by the remainder of the distribution (which may be considered noise, using that term in a broad sense) is significantly higher for (*b*) than (*a*).

The data model is required to yield the expected null hypothesis behaviour of a uniform U(0,1) output when drawing random samples. The model should also retain information about relative amplitudes, and hence the effective signal-to-noise ratio. The significance of this property can be explained using figure 6, where amplitude distributions for two rings of *resel* samples are shown. The model applied should reflect the differences between the two distributions, even when assessing the highest amplitude sample in each, and despite the amplitudes (and probability mass) of those samples being equal. Intuitively, the probability that the highest sample can be explained by the remainder of the distribution is lower for figure 6*a* than figure 6*b*, as in the former there is significant separation between that sample and the remaining distribution, unlike in the latter.

Satisfying both requirements simultaneously is difficult, but the model adopted offers a very good compromise between objectives, exploiting the fact that the sensitivity to relative amplitudes creates the greatest concern at the highest amplitudes where the signal most likely reflects a cause other than noise; however, the vast majority of samples will lie well below such extreme values. This majority of samples, up to a high amplitude threshold, can be fitted to a discrete distribution to assure conformity with the expected output under the null hypothesis, and the above-threshold samples can be represented separately, fitting a suitable analytic distribution to this tail. This sort of tail fitting can be facilitated by a generalized *Pareto* distribution [48,49]. The c.d.f. of such a distribution is specified by

3.1 $$F_{\xi ,\sigma }(x)=\left\{ \begin{array}{ll} 1-{\left(1+\frac{\xi x}{\sigma }\right)}^{-1/\xi } & \text{for}\;\xi \neq 0\\ 1-\exp\left(-\frac{x}{\sigma }\right) & \text{for }\;\xi =0, \end{array}\right.$$

where *ξ* and *σ* are parameters of the distribution, and *σ*>0, *x*≥0 if *ξ*≥0, but 0≤*x*≤−*σ*/*ξ* if *ξ*<0.

**Figure 7.**
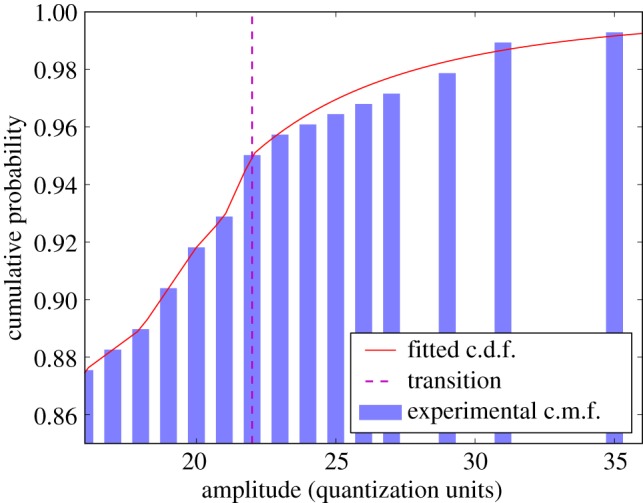
A semi-parametric model applied to the cumulative version of the distribution presented in figure 6*a*, plotted over a limited domain. This reveals the key feature of the semi-parametric data model: up to the transition threshold the fitted distribution exactly matches the discrete, piecewise-linear fit, but above a *Pareto* distribution is fitted to the upper tail of the c.m.f. This is seen as a smooth curve that no longer exactly matches the tops of the bars. The *p*-value at the very highest amplitude recorded is nearly an order of magnitude lower than in the equivalent purely discrete model.

An example of this semi-parametric model is shown in figure 7. The determination of the threshold (indicated by the dashed transition line in figure) at which to transition from one part of the model to the other can be critical, as setting the threshold too low invalidates the tail fit, but an excessively high threshold leaves very few points against which to map the continuous distribution—here it is broadly set to capture the top 5% of the samples in the tail. Imposing a continuity constraint on the fitted curve at the transition point from the discrete distribution reduces the number of parameters to solve for in a maximum-likelihood optimization.

**Figure 8.**
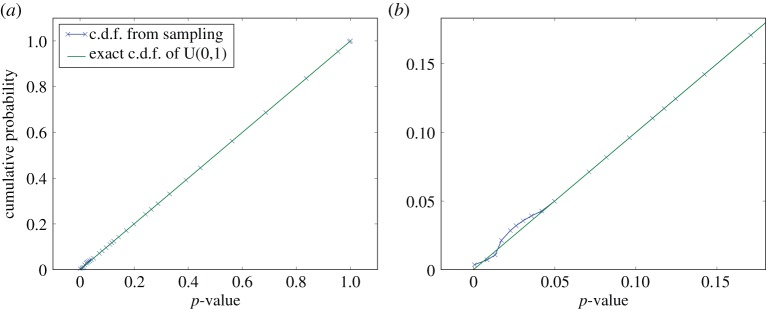
The c.d.f.s for the output of the semi-parametric model shown in figure 7. The full set of amplitudes that were used to generate the model were converted to *p*-values using the model, and the c.d.f. was determined for these. Plot (*a*) shows the full domain, while (*b*) a zoomed section at the origin. As should be expected for a discrete model, the samples follow the continuous U(0,1) c.d.f. exactly, except at the very lowest *p*-values (corresponding to especially high amplitudes) where there is a slight deviation, the result of the *Pareto* tail-fit.

In principle, such a distribution provides the previously explained sensitivity to relative amplitudes in the domain of interest (the very highest amplitudes) that is sought at minimal cost to the validity of a uniform U(0,1) expected output under the null hypothesis. The two distributions of figure 6 allow the sensitivity to relative amplitudes to be demonstrated by computing the *p*-value of the highest amplitude. This is done by forming the model c.d.f. from the local *resel* amplitude distribution and then subtracting the c.d.f. value at the evaluation amplitude from unity. Applying a purely discrete model to the two plotted distributions yields 3.6×10^−3^ in each case. Switching to the semi-parametric model gives the *p*-values 5.5×10^−4^ and 9.3×10^−3^, for the distributions of figure 6*a*,*b*, respectively. Thus, the fact that the amplitude in question is better separated from the remainder of the distribution in the former than the latter distribution is reflected in the *p*-values—the value for the former is more than an order of magnitude lower than for the latter. The result of testing the behaviour of the model under *H*_0_ is shown in figure 8, where the match to the expected output distribution is perfect apart from a slight deviation at the very lowest *p*-values (corresponding to especially high amplitudes). This deviation is considered negligible, as is confirmed later. This semi-parametric model has therefore been demonstrated to provide the desired sensitivity to effective signal-to-noise ratio (at the highest amplitudes, where this matters) at negligible cost to the validity of the null hypothesis expected output.

Note that this semi-parametric, local model makes no attempt to explicitly separate noise and flaw contributions in the received signal. The model is based simply on drawing attention to the most extreme amplitudes, which are the most likely to be the result of something other than noise, and exploits the fact that indications will only occupy a tiny fraction of the data volume, and hence *resels*. This data model will from now on be termed the local empirical noise (LEN) model. It is used to convert all channels' *resel* amplitudes to *p*-values. How these *p*-values should be combined is the subject of the next section.

### (d) Combing *p*-values: consensus test

To combine *p*-values relating to different independent tests there are so-called consensus, or family-wide, *p*-value tests available, all based on the fact that under the null hypothesis the *p*-values should be random samples from the uniform distribution U(0,1) distribution. The general procedure underlying these consensus, family-wide, *p*-value tests is to form a test statistic from the *p*-value inputs, and then map that test statistic value to a (single) output *p*-value by invoking the c.d.f., or more exactly s.f., of the test statistic. Conceptually, there can be multiple different consensus tests, all of which under *H*_0_ receive as input multiple random samples from U(0,1) and map these onto a single U(0,1) output, because this requirement still leaves freedom of choice in the weighting of inputs. For example, in some tests *p*-values are able to ‘cancel symmetrically’ about the 0.5 distribution mid-point (so that e.g. combining *p*-values 0.3 and 0.7 would yield 0.5), whereas in others low *p*-values are permitted more influence than the corresponding high values. For the data processing to be conservative, the test chosen must be of the latter sort, and not allow a single low *p*-value to be cancelled out in effect by an ‘opposite but equal’ high *p*-value in the consensus test input. Note that AND and OR logical combinations bound the range of possible combination methods (see also Results, §4*a*) [46].

One such test, widely used elsewhere and applied here is known as *Fisher's combined probability test*, or *Fisher's method* [50]. The test forms a test statistic from the sum of the natural logs of the *p*-values in the collection [51]

3.2 $$s_{k}=-2\sum_{i=1}^{k}\ln(q_{i}),$$

where *s*_*k*_ is the test statistic for *k* *p*-value inputs *q*_*i*_. Under the null hypothesis, this quantity follows a *χ*^2^ distribution with 2*k* degrees of freedom, $s_{k}∼\chi_{2k}^{2}$, so the s.f. appropriate to that *χ*^2^ distribution may be used to convert the test statistic value to an output *p*-value *q*. The explanation for this is that twice the negative logarithm of a U(0,1) input follows a *χ*^2^ distribution, and the sum of independent *χ*^2^ variables is itself *χ*^2^, with a number of degrees of freedom equal to the total of the inputs' degrees of freedom [52].

#### (i) Discrete data and the *p*-value discretization problem

**Figure 9.**
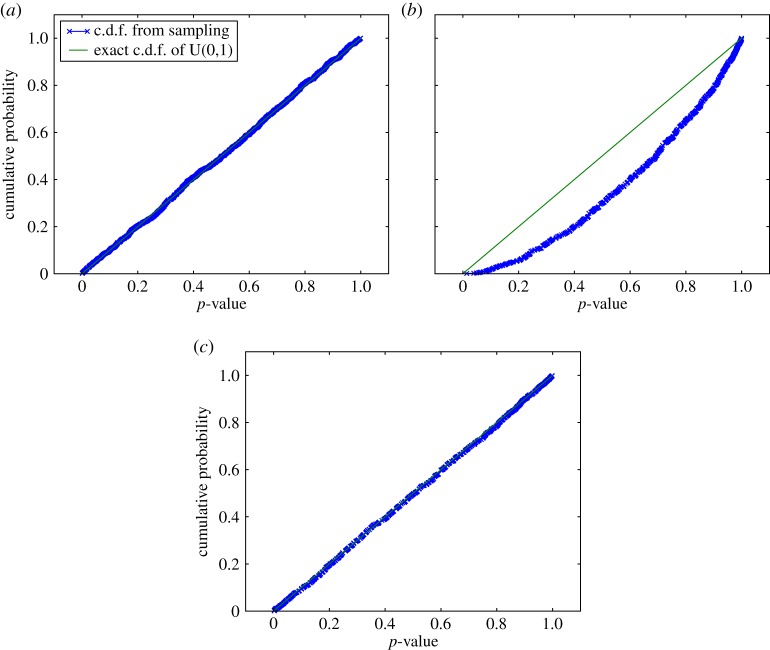
C.d.f.s for the output *p*-value of the *Fisher* consensus test for different simulated inputs, compared against the theoretically expected. In (*a*) 5 *p*-values drawn (by *Monte Carlo* (MC) sampling, simulating null hypothesis conditions) from the continuous U(0,1) distribution (see figure 5*a*) are combined, and the experimental distribution closely matches the theoretical. Plot (*b*) was produced by drawing two samples from the discrete distribution of figures 5*b* and three from that in figure 5*c*, and a large deviation from the continuous U(0,1) c.d.f. is observed, as is typical for discrete test inputs. In plot (*c*) the same inputs are processed as in (*b*), but this time including compensation for discretization, corresponding to the MC-based evaluation of *Fisher's exact test*. Here the experimental distribution is seen to closely correspond to the continuous U(0,1) distribution, demonstrating the effectiveness of the discretization compensation.

The discussion of consensus testing has, at least implicitly, assumed that the *p*-value inputs are samples from the continuous U(0,1) distribution under the null hypothesis, rather than from any discrete variant. However, the *p*-values that result from the application of the local, semi-parametric model (developed in §3*c*) to convert *resel* amplitudes to probabilities will be discrete. Not only that, but different *p*-values originating from different local distributions will be discretized differently, in general, with different numbers of irregularly spaced levels available. Naively applying the consensus tests as described to discrete, rather than continuous, *p*-value inputs causes some remarkable, problematic distortions, illustrated in figure 9*a*,*b*. Here, the output of the *Fisher* consensus test is examined, presented as a c.d.f. of consensus *p*-values, for inputs drawn from the different U(0,1) distributions presented previously, in figure 5. While the continuous input case closely matches the expected null-hypothesis behaviour of a U(0,1) output, for discrete *p*-value inputs severe deviations from this occur, as seen in the typical example of figure 9*b*.

In practice, such distortions render the test unusable for typical discrete inputs, as even under the null hypothesis the probability of a significant result substantially differs from the significance level. For example a *p*-value of 0.2 has an associated cumulative probability of about 0.06 rather than 0.2 as would be expected. While it is possible that the effects observed for discrete inputs are not a concern in the sort of meta-studies in which these consensus tests are typically applied as the input *p*-values are always suitably continuous, in principle, this is a problem relevant to a wide range of situations, bearing in mind that all digital data are quantized. This problem has parallels to the so-called familywise error rate in conventional multiple testing. Because of the large number of individual hypothesis tests being considered together, there is a high probability of falsely rejecting the universal *H*_0_, that none of the tests are significant. Adjustment procedures exist, the most common of which is the *Bonferroni* correction that scales down the significance level to be applied to individual tests by the number of such tests considered together [30,53]. Alternatively, the individual *p*-values themselves may be adjusted [54].

#### (ii) Discretization compensation

The observed deviations from the required null hypothesis behaviour (as in figure 9*b*) were investigated further by studying the intermediate stage of the consensus testing process, the test statistic and its s.f., for the problematic, discrete input cases. The discretization of the input *p*-values was found to make the s.f. of the test statistic diverge from that expected for continuous inputs, a *χ*^2^ distribution in the case of the *Fisher* test. To compensate for this, several compensation possibilities were explored. The approach ultimately adopted consists of applying the actual, discrete s.f. rather than the s.f. expected from continuous statistics theory. In practice, this discrete s.f. is approximated from MC sampling, by computing the test statistic many times using MC samples drawn from the discrete input *p*-value distributions—in effect simulating the expected null hypothesis behaviour. This s.f. can readily be computed *a priori* for any discrete inputs and choice of test statistic, although while the continuous *Fisher* test s.f. only relies on the number of *p*-values in the input, the MC scheme requires knowledge of all possible discretization levels that each input *p*-value could take, as provided by the local data model. The effectiveness of the scheme is demonstrated by figure 9*c*, where the plot showing the poor null hypothesis behaviour (figure 9*b*) is reproduced but with the application of the MC s.f.s: the distortions previously observed have been removed. The approach corresponds to the evaluation of *Fisher's exact test*, typically used in the context of the so-called contingency tables [55]. Moreover, this is based on the hypergeometric distribution, which is known to be hard to evaluate and a range of tools have been used to tackle it, including MC methods [54,56].

The quantity that directly links the accuracy of the MC evaluation and the computational effort required to achieve it is the number of samples used. Typical MC theory states that the former improves proportional to the reciprocal of the square root of this sample number [57]. This relationship has been confirmed to apply also here. There are a number of computational approaches to reduce the computing time required to give a certain accuracy. A simple option is to perform the evaluations in parallel, using several CPU cores simultaneously [58]. A numerical technique to give consistent coverage of the space being explored by random sampling, and hence potentially reduce the number of samples needed for a given accuracy, is to draw in the inputs from a low-discrepancy sequence spanning the [0,1] interval. These pseudo-random sequences avoid random clustering of samples that would occur with a more conventional random number generator, and the *Sobol* sequence is a commonly used example [57,59]. A more complex technique is importance sampling: this involves biasing the used samples to the part of the [0,1] interval where they provide the greatest value in the output, and then compensating for the biasing in the collation of the output [57]. Because in practice the consensus *p*-values of indication region *resels* will usually be very low, the resolution of the s.f. for that consensus *p*-value range is critical to be able to distinguish between regions at all. Achieving the required accuracy by unbiased MC sampling is very wasteful, but this problem can readily be addressed by the application of importance sampling [38].

#### (iii) Combining consensus test and semi-parametric model

**Figure 10.**
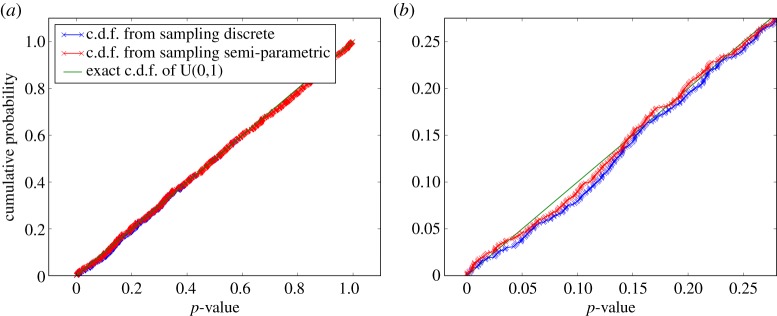
The output c.d.f.s for combining the semi-parametric data model with MC-based evaluation of *Fisher's exact test*, applied to experimental data. The plots are based on the amplitude distribution found in one circumferential ring of *resel* data (figure 6*a*). Sets of five *p*-values were obtained using both the (exact) discrete and the semi-parametric models, then these were passed through the MC compensated *Fisher* consensus test to build up distributions for the output consensus *p*-values. The only underlying difference between the curves shown is the choice of data model. In (*a*), no difference is discernible between the two curves, only in the zoomed plot (*b*) is the effect of the semi-parametric model's tail fit identifiable.

As a final test of the system, the behaviour of the consensus output when operating on inputs generated by the semi-parametric data model (see §3*c*) applied to experimental data were studied. The resultant c.d.f.s for such trials are shown in figure 10. These both demonstrate the effectiveness of the consensus test compensation and provide evidence in support of the earlier assertion that the deviation from the null hypothesis behaviour introduced by the semi-parametric model is negligible in practice. It is noteworthy though that the induced deviation increases with the number of *p*-values combined in the consensus test and will also increase with the magnitude of the semi-parametric tail fit error. If such a deviation is not acceptable, the purely discrete data model should be relied upon instead of the semi-parametric model proposed.

#### (iv) Comparing consensus test outputs

Having used the described probabilistic data fusion system to assign a consensus *p*-value to all *resels* in the multichannel dataset, we wish to compare and rank these values. Importantly, all the fused *p*-values, while strictly discrete, are in practice samples of the continuous uniform U(0,1) distribution to a very close approximation. This is related to the fact that the number of possible unique input combinations from the contributing distributions increases approximately exponentially with the number of signals being combined.

#### (v) Summary of data fusion method

**Figure 11.**
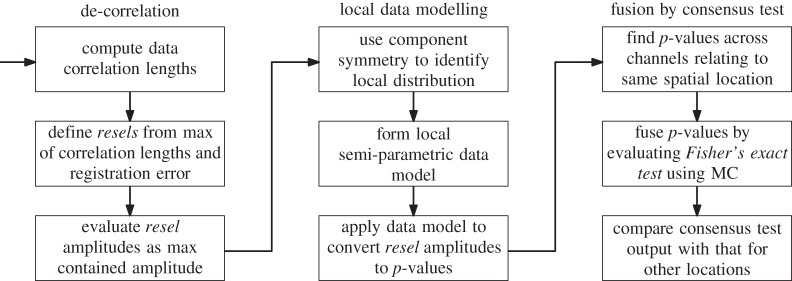
The data fusion method summarized as a flow diagram. Note that there are several important processing steps that precede those shown here, notably registration (see §2) and channel-specific processing.

A schematic summary of the data fusion approach developed and described in the preceding subsections is provided by the flow diagram in figure 11.

## 4. Results

Using the DFD, a consensus *p*-value may be assigned to any spatial region of interest in the data volume. Subtracting this value from unity gives a probability of greater intuitive appeal, increasing for less noise-like regions. This is termed the indication severity probability. While this value is expected to be displayed for an operator to see when he or she is reviewing the sequence of suggested indications, it should be emphasized that this should only be used as a rough guide. The decision of when to terminate the analysis must be made by the operator based only on the viewed signals, and therefore for instance no hard threshold (equivalent to a significance level for the *p*-value) should be set *a priori*.

The results here are presented in terms of the POD and PFA or more specifically the ROC that relates the two. These quantities can be computed for the data fusion output given some test data with known indications. Typical calculations of POD and PFA rely on computing integrals of distributions *p*(*signal*|*flaw*) and *p*(*signal*|*noise*) for a given amplitude threshold, which may be varied to build up an ROC curve [9–11]. Here the process is similar, but completed in terms of indication severity probability rather than signal amplitude, with a chosen significance level taking the place of the amplitude threshold.

The noise distribution, *p*(*severity*|*noise*), is built up by dividing the entire component volume into candidate indication regions and computing indication severity probabilities for each. The implementation of this full evaluation is non-trivial, and not only because of the sheer computational load and the fact that care should be taken to omit the regions containing the known indications. Rather, the major challenge concerns the decision of which *p*-values to fuse, given that the originating data channels are described in terms of aligned coordinate axes (post-registration) but different coordinates. Essentially, in each channel physical space is discretized differently, and in a somewhat arbitrary manner, such that determining associations between *resels* requires some approximation [39].

The flaw distribution, *p*(*severity*|*flaw*), ideally requires a collection of indication severity probabilities to be available for a number of indication regions considered to contain equivalent defects. However, often only a single such indication severity probability is available, which is then used to create a distribution empirically. This is done here by making the assumption that the amplitudes from the defect vary like the local noise distribution, creating for each contributing *resel* a duplicate of the local noise distribution with the mean shifted to the value of the *resel*.

As will become clear in the results that follow, moving from simple amplitude distributions to the consensus (indication severity) probability space that incorporates knowledge of local data distributions and correlations between independent samples of the same spatial region allows substantially improved separation of noise and defect signals to be achieved.

### (a) Qualitative performance

Here, we compare the DFD with the standard logical AND and OR operators that effectively provide bounding fusion possibilities, to gain a qualitative understanding of its performance. Taking the indication severity probability (unity minus a consensus *p*-value) to correspond to a ‘probability of success', we apply binomial theory to compute the probability of obtaining ‘all successes’ and ‘at least one success’, for AND and OR, respectively. Thus, the AND consensus test output *q*_AND_ for *N* input *p*-values *q*_*i*_ is then

4.1 $$q_{\mathrm{AND}}=1-\prod_{i=1}^{N}(1-q_{i}).$$

The corresponding OR consensus test output *q*_OR_ is

4.2 $$q_{\mathrm{OR}}=\prod_{i=1}^{N}q_{i}.$$

**Figure 12.**
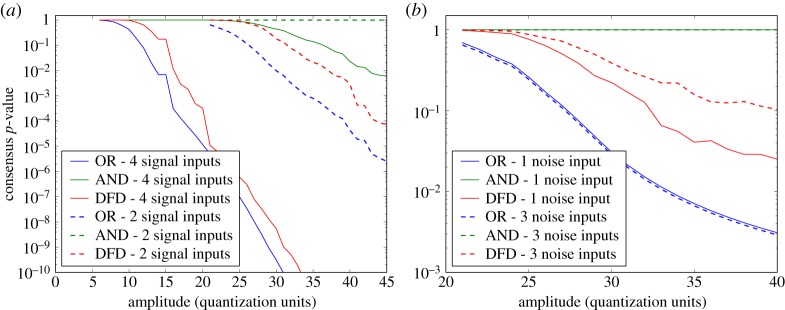
A comparison the DFD compared with AND and OR operators, acting on the same *p*-value inputs. In (*a*), the detectors receive four or two inputs of the same amplitude (but differing *p*-values, as drawn from different distributions). Qualitatively, the DFD is seen to transition from behaving like an AND test to behaving like an OR test as the amplitude increases. The fall in consensus *p*-value output is less pronounced and delayed to higher amplitudes if only 2 rather than 4 inputs are provided. In (*b*), a single input of the varied amplitude is corrupted by either 1 or 3 noise-like inputs. The DFD is seen to not reach as low outputs when more noise is added, whereas AND and OR combinations are almost insensitive to the increase plotted.

Figure 12*a* shows how the three detectors vary when provided with four or two inputs of the same amplitude (but differing *p*-values, as drawn from different *resel* distributions of the industrial dataset). Considering the four input plots first, as should be expected, all three detectors give a decreasing output consensus *p*-value as the amplitude increases, and the OR consensus test output falls off at a lower amplitude and more rapidly than the AND consensus test. The DFD is seen to transition from behaving like an AND test to behaving like an OR test as the amplitude increases. This is (qualitatively) ideal behaviour for a detector, as the AND-like behaviour is desirable at low amplitude to suppress the false-call rate, yet OR-like behaviour is required at higher amplitudes so as to maintain sensitivity to amplitude spikes in a single input. The exact balance and transition between these two states is governed by the specific implementation of the data fusion, for example in the tail fit of the semi-parametric data model, and is open for debate and adjustment. If only two rather than four inputs are provided, similar trends are observed, but for all tests the fall in consensus *p*-value output is less pronounced and delayed to higher amplitudes. This can rationalized as four defect signals provide greater confidence of the presence of a defect than just two defect signals at the same amplitude.

Figure 12*b* compares the three detectors in a different test scenario: a single input *p*-value resulting from the varied amplitude is effectively corrupted by either 1 or 3 noise-like inputs. Again, the DFD is seen to behave as an intermediate between AND and OR consensus tests, and a transition from AND-like behaviour to more OR-like behaviour is visible as the amplitude increases. Note that the AND consensus test output does not vary as that test is undermined by any noise inputs—potentially dangerous in practice, as if any channel does not contain a significant defect response (for instance, due to the defect reflectivity being highly directional, or maybe a break-down in probe coupling) the defect would be missed. As the number of noise inputs is increased, the DFD performance is reduced but not completely undermined, while the other two tests are essentially unaffected. This shows that the DFD has some resilience to corrupting noise inputs, but the reduction in sensitivity does mean that if there is prior knowledge that a defect response will be very poor in a particular channel, for example due to the directionality of the defect or probe near-field effects, that channel should be manually excluded from the fusion process for improved detection performance.

### (b) Quantitative performance on an industrial dataset

This section considers results from processing data from the inspection of a Rolls-Royce seeded defect disc, containing realistic defects after having been forged from contaminated billet. The data acquisition followed the current standard inspection procedure and no supplementary checks were made on the data quality. The disc inspected has five known point-like defects from contaminant inclusions, identified by traditional ultrasonic and radiographic methods, including an ultrasonic amplitude threshold detector (ATD), with a threshold set in line with the currently deployed Rolls-Royce inspection procedure (at 64 amplitude quantization samples). A schematic diagram, including approximate locations of the known indications considered here, was provided in figure 1*b*, and the coverage map showing the data fusion opportunities was shown in figure 3.

#### (i) Known Indication 1

**Figure 13.**
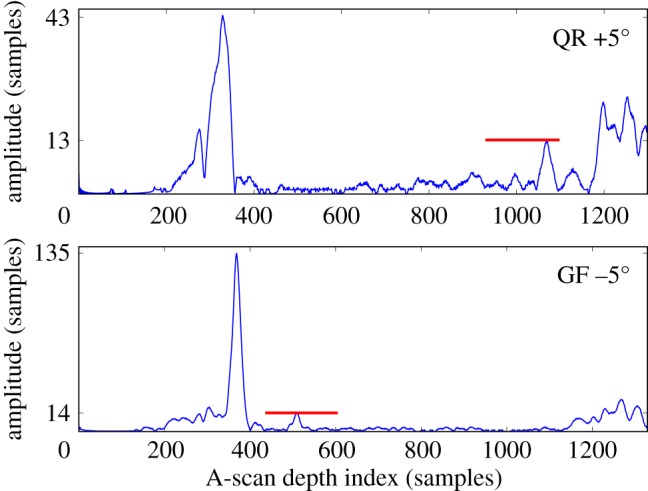
Two envelope-detected A-scans for the location of the first known indication, focusing on the channels (as labelled in terms of the scanned surface of the disc cross section seen in figure 1*b* and probe angle) recording the lowest amplitude in that *resel*. The red lines mark the extent and amplitude of the *resel* in question. These *resel* amplitudes are very low compared with the currently used global amplitude threshold at 64 amplitude samples. Front and back surface echoes are seen in both A-scans around 350 and 1200 A-scan samples, respectively.

**Figure 14.**
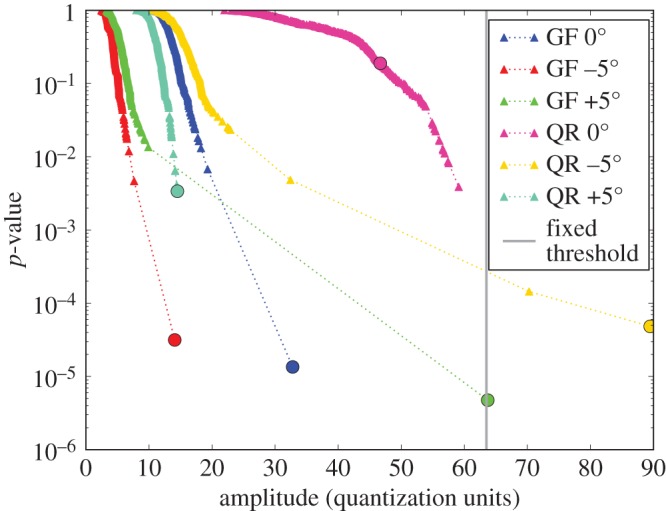
Converting the *resel* amplitude distributions contributing to the location of the first known indication to LEN *p*-values. The *p*-value in each channel at the *resel* of the known indication is marked by a circle—only two of these are picked up by the presently used fixed threshold. The signals from the A-scans in figure 13 are the left-most circles. Note that while these amplitudes are significantly below the fixed threshold of the currently used ATD, they are the highest amplitudes within their own distribution, so that they have low *p*-values. The legend identifies each channel in terms of the surface of the cross section scanned (figure 1*b*) and the probe angle to the surface normal.

First, we consider the first known indication, considered to be the easiest to find. This indication is seen in six scans. The A-scans for two of the scans, picking the two in which the amplitude in the indication *resel* is lowest, are shown in figure 13. While a reflection from the indication is identifiable, the amplitude is very low. A more useful presentation of the data to demonstrate the workings of the DFD is provided by figure 14, which shows how in each data channel the amplitude of the *resel* at the indication is converted to an LEN *p*-value using the local distribution of *resel* amplitudes. Note that the low amplitudes seen in the A-scans are the highest amplitudes in the local distributions so are converted to very low *p*-values.

**Figure 15.**
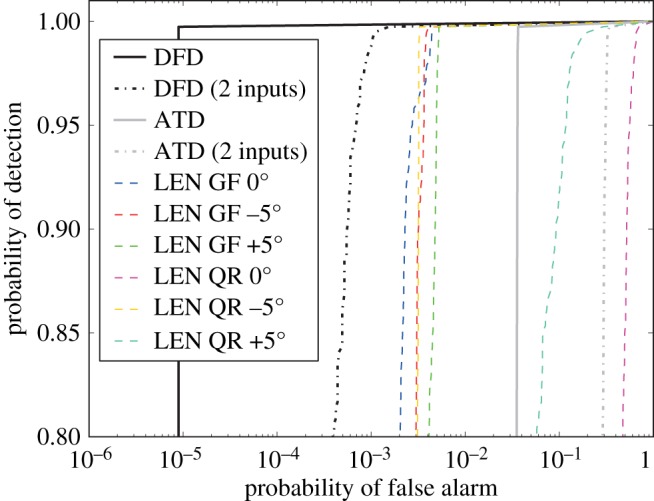
The ROC curves for the first known indication of the disc. This indication is seen in six scans, four of which provide an LEN detector superior to the conventional ATD. But the DFD comprehensively outperforms all of the inputs—and in fact provides an essentially perfect detector, as the probability of false alarm value reached is as low as can be numerically estimated from the available data. Also shown is the DFD and ATD considering just two inputs, the QR +5^°^ and GF −5^°^ scans seen in figure 13. Despite the underlying amplitudes being very low, even this limited fusion offers a great improvement over not only the ATD for the two inputs, but also the ATD for all inputs.

ROC curves may then be calculated as shown in figure 15. This plot features curves for the DFD and ATD operating on all six channels, as well as on just the channels considered in the A-scan plot, figure 13, and the curves for the LEN detector of each individual channel, that relies on the local data model conversion to *p*-values without subsequent fusion. Focusing on the DFD and ATD curves for all channels, four scans are seen to provide an LEN detector already superior to the conventional ATD—but the DFD comprehensively outperforms all of the inputs. In fact, it provides an essentially perfect detector, as the PFA reached at optimal POD is as low as can be numerically estimated from the available data, more than three orders of magnitude lower than that of the ATD. Even with just two inputs, those of the lowest *resel* amplitude, the DFD is seen to outperform either ATD ROC plotted.

#### (ii) Known indication 4

**Figure 16.**
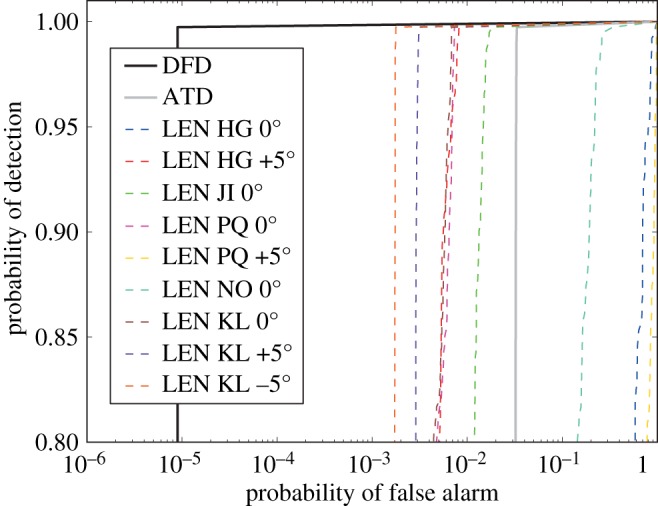
The ROC curves associated with the fourth known indication of the disc. This location is inspected by nine scans, five of which provide an LEN detector that is already better than the ATD. Combining all the LEN inputs in the DFD again gives a very substantial further improvement.

Now we consider the ROC curves for the fourth known indications, one that is harder to find than the first examined. For this location nine scans contribute, and the final results are shown in figure 16. Five scans provide an LEN detector that is better than the ATD. Combining all the LEN inputs in the DFD again gives a very substantial further improvement, lowering the PFA by over two orders of magnitude compared with even the best of the LEN detectors (the one for the KL −5^°^ scan). The DFD performance has thus further been verified.

#### (iii) Overall

It is also noteworthy that the five *resels* of the lowest consensus *p*-value output, and hence the highest indication severity probability, also correspond exactly to the five known indications in the disc. Therefore, if the DFD were deployed in this inspection as proposed, with a human inspector reviewing *resel* locations sequentially, as ranked by decreasing indication severity probability, the known indications would be immediately identified. But in such a scenario, the human inspector may not stop at the fifth *resel*. Given the contaminated billet used in this disc may have also given rise to further, as-yet undetected defects, it may thus be possible to locate defect indications beyond the capabilities of previously used inspection approaches. In fact, several more indications have been found but these have not yet been independently confirmed to be defects [38]. Moreover, a further speed-optimized version of the program could possibly provide initial results while the component is still being scanned, allowing suspect regions to be revisited for further interrogation.

## 5. Conclusion

The paper has described a novel yet general process by which samples from diverse amplitude fields may be fused to form a single probability, appropriate for evaluation of large, systematically sampled amplitude fields that overlap spatially, as is seen in automated NDE. The basic processing stages are data de-correlation, local data modelling to convert amplitudes to probabilities taking into account local data statistics, then fusion of the probabilities for a spatial region of interest using a consensus test. The processing scheme is essentially free of arbitrary thresholds, makes very few assumptions, and is adaptable to a range of applications. Novel elements include the semi-parametric data model and the application of *Fisher's exact test*. The authors are not aware of a comparable data fusion system having been developed elsewhere, in any field, and the work described here may have applications beyond NDE. This system was shown to provide qualitatively ideal behaviour for a fusion system. Tests of the system on an industrial dataset convincingly demonstrated the power of the developed DFD over conventional detection approaches, such as those currently used in industry. The DFD can be used not only to improve POD and/or PFA for defects detectable by more conventional means, but also potentially to enable the detection of smaller defects at a PFA still acceptable in practical use.
